# Rainfall seasonality on the Indian subcontinent during the Cretaceous greenhouse

**DOI:** 10.1038/s41598-018-26272-0

**Published:** 2018-05-31

**Authors:** Prosenjit Ghosh, K. Prasanna, Yogaraj Banerjee, Ian S. Williams, Michael K. Gagan, Atanu Chaudhuri, Satyam Suwas

**Affiliations:** 1 Centre for Earth Sciences, Indian Institute of Science, Bangalore, 560012 India; 2 Divecha Centre for Climate Change, Indian Institute of Science, Bangalore, 560012 India; 3 Birbal Sahni Institute of Palaeosciences, 53, University Road, Lucknow, 226007 India; 4 Research School of Earth Sciences, The Australian National University, Acton, ACT 2601 Australia; 5 School of Earth and Environmental Sciences, The University of Queensland, Brisbane, QLD 4072 Australia; 6 Department of Materials Engineering, Indian Institute of Science, Bangalore, 560012 India

## Abstract

The Cretaceous greenhouse climate was accompanied by major changes in Earth’s hydrological cycle, but seasonally resolved hydroclimatic reconstructions for this anomalously warm period are rare. We measured the δ^18^O and CO_2_ clumped isotope Δ_47_ of the seasonal growth bands in carbonate shells of the mollusc *Villorita cyprinoides* (Black Clam) growing in the Cochin estuary, in southern India. These tandem records accurately reconstruct seasonal changes in sea surface temperature (SST) and seawater δ^18^O, allowing us to document freshwater discharge into the estuary, and make inferences about rainfall amount. The same analytical approach was applied to well-preserved fossil remains of the Cretaceous (Early Maastrichtian) mollusc *Phygraea (Phygraea) vesicularis* from the nearby Kallankuruchchi Formation in the Cauvery Basin of southern India. The palaeoenvironmental record shows that, unlike present-day India, where summer rainfall predominates, most rainfall in Cretaceous India occurred in winter. During the Early Maastrichtian, the Indian plate was positioned at ~30°S latitude, where present-day rainfall and storm activity is also concentrated in winter. The good match of the Cretaceous climate and present-day climate at ~30°S suggests that the large-scale atmospheric circulation and seasonal hydroclimate patterns were similar to, although probably more intense than, those at present.

## Introduction

One consequence of global warming is predicted to be an increase in the frequency and intensity of extreme weather events, including large storms^1^. Palaeoclimate records of the size and frequency of storms during past periods of sustained high global temperatures are essential for testing this prediction, but such datasets are rare and difficult to extract from the geological record. In present-day India, tropical storms and cyclones are associated with increased atmospheric convection during the summer monsoon. This activity brings large amounts of fresh water to the sub-continent and increases river discharge, reducing the salinity of coastal waters. Seasonal variation in temperature and rainfall are important factors that are likely to be affected by rising levels of atmospheric CO_2_^2^. There is strong evidence for a shift in the seasonal distribution and inter-annual variability of precipitation worldwide, particularly at higher latitudes, as atmospheric *p*CO_2_ has increased over the last century^1^.

The Cretaceous was a period of global greenhouse conditions when atmospheric *p*CO_2_ was at least three times the present level^3^. This period saw one of the largest-known increases in sea level and global temperature, factors which may have been responsible for major changes in the hydrological cycle^4^. A record of expansion of the Hadley circulation and seasonal variations in wind strength is preserved in high latitude sedimentary deposits^5^. Palaeoclimate modelling indicates elevated humidity and rainfall at mid-latitudes of the southern hemisphere, although identifying the source of the moisture for regional precipitation remains problematic^6^. Proxy records of temperature and rainfall seasonality during the Cretaceous potentially can provide insight into the conditions to be expected in the future should the modern trend in global warming continue.

**Figure 1:**
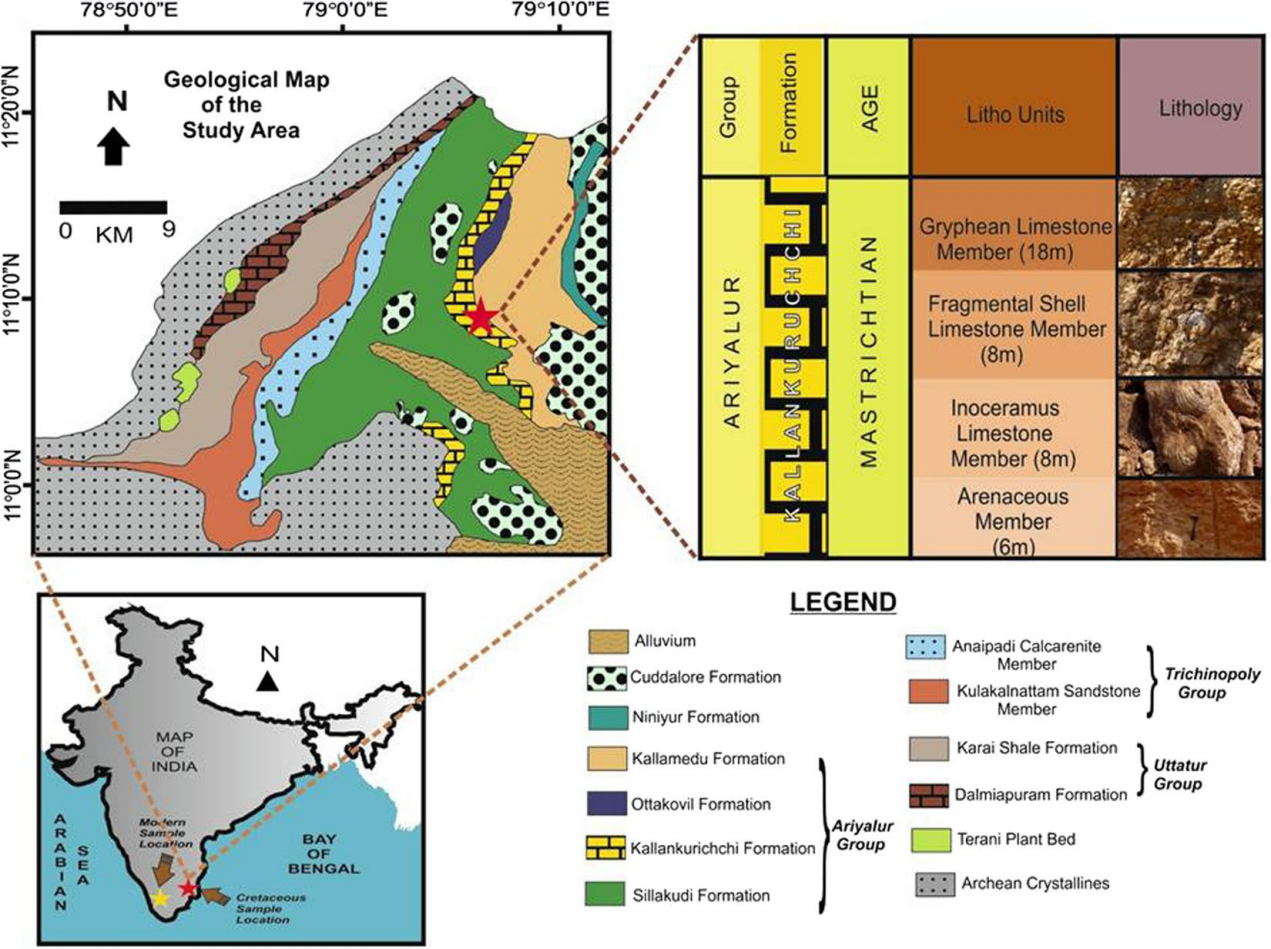
Location of the study areas. (**A**) Map of India showing the location of the modern Cochin estuary (yellow star) and Late Cretaceous Cauvery Basin (red star). (**B**) Geological map of the Cauvery Basin showing the sampling location and lithostratigraphy of the Late Cretaceous succession. Maps were drawn using CorelDRAW Graphics Suite (2017) Education License, Graphic design software, https://www.coreldraw.com/.

During the Late Cretaceous, the Indian plate was located about 30° south of the equator. A history of Cretaceous sedimentation at the south-eastern margin of the Indian continent is well preserved in the Cauvery Basin (Fig. 1), an elongated pericratonic rift basin extending from south-eastern India into western Sri Lanka^7–9^. Sandstones of Campanian age are overlain by Early Maastrichtian limestone of the Kallankuruchchi Formation, which contains shells of molluscs such as *Phygrea sp*. and *Gryphaea sp*.^10,11^. It has been argued, based on the presence of hummocky cross stratification, that the sediments containing the shells were transported from a nearby shelf region and re-deposited as a result of storm activity^10^.

Some well-preserved shells with original mother-of-pearl nacreous layers have alternating dark and light growth bands, reflecting growth under relatively quiet and turbulent sea conditions, respectively. These growth bands record seasonal changes in the isotopic composition of the sea water, reflecting changes in the discharge of freshwater from local rivers^12^ (Suppl. Fig. S3). In combination, the δ^18^O and CO_2_ clumped isotope (Δ_47_) signatures in the carbonate growth bands carry information about fluctuations in both the water temperature and freshwater input into the shallow estuary^13^ in which the molluscs grew.

The contribution of freshwater to an estuarine system is governed by rainfall amount in the local catchment. The salinity and δ^18^O of estuarine water, therefore, is expected to covary with precipitation. In the first part of our study, we show that measurements of δ^18^O and Δ_47_ in seasonal growth bands in modern shells of the Black Clam (*Villorita cyprinoides*; growth rate ~0.75 mm/month, Suppl. Fig. S1a) accurately record shifts in the δ^18^O of estuarine water that directly reflects measured seasonal variation in local freshwater discharge. Estimates of seasonal freshwater discharge obtained from the isotopic records (Suppl. Fig. S4) are closely correlated with the measured cumulative discharge from the major regional river feeding the estuary (the Periyar River) for the year 2008–2009^14^. Seasonal changes in rainfall measured at the Wellington Island meteorological station, near Cochin, also correlate strongly with the river discharge data^15^. Based on this result, we then apply the same analytical approach to well-preserved mollusc shells from the Early Maastrichtian sedimentary succession in the nearby Cauvery Basin. The new palaeoenvironmental record provides insight into rainfall seasonality and freshwater discharge in southern India during the hothouse conditions that prevailed in the Late Cretaceous.

## Results

### Reconstruction of freshwater discharge

Studies of the *V. cyprinoides* from the coastal region of Cochin, Kerala (Fig. 1), show that the growth rate of their shells approaches a minimum of 0.25 mm/month during cooler drier months, when the salinity of estuarine water is at a maximum, and peaks at >1.3 mm/month during warmer wetter months, when freshwater discharge lowers salinity^16,17^.

**Figure 2:**
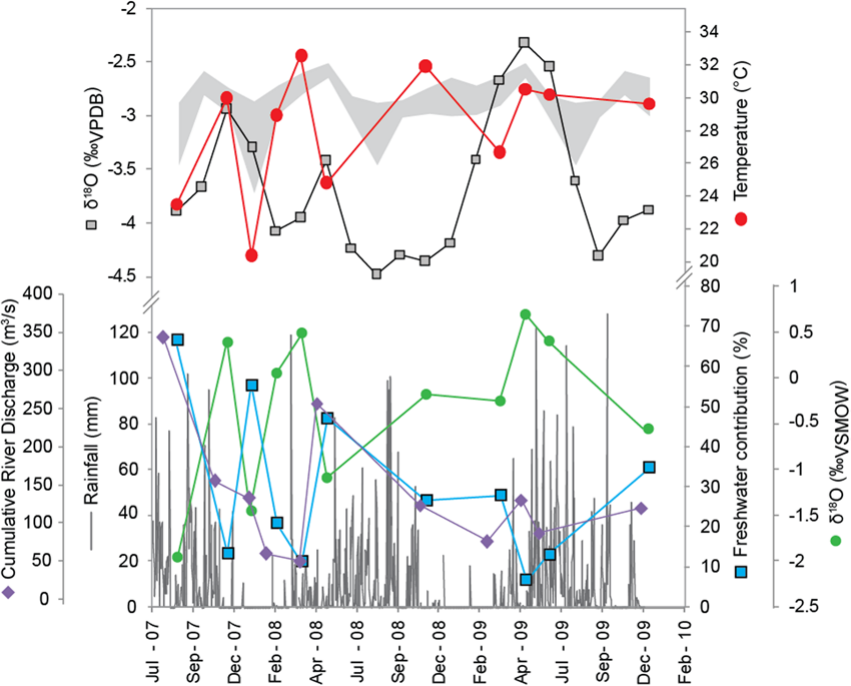
δ^18^O in *V. cyprinoides*, reconstructed temperatures and instrumental records for the Cochin estuary. (**A**) Temperature estimates (red circles) derived from clumped isotope thermometry (CDES scale) compared with observed temperatures^45^ (grey shading denotes amplitude of signal). δ^18^O values for *V. cyprinoides* measured by continuous flow IRMS at 1 mm resolution (grey squares) are also plotted. Slower shell growth during cooler periods reduced the temporal resolution for the temperature estimates. (**B**) Observed daily rainfall (grey bars, http://www.tutiempo.net/) compared with the reconstructed percentage contribution of freshwater to the Cochin estuary (blue squares) and cumulative river discharge (purple diamonds, discussed in detail in Section S4 of the Suppl. Information 1). The freshwater contribution was calculated by subtracting the influence of temperature from the shell δ^18^O record, and applying a mixing model with end-member δ^18^O values for freshwater and seawater (green circles denote calculated δ^18^O water) (see text for details).

The shell isotopic data reported here are from the first ~2.5 years of the life of a Black Clam collected live from Thevara, ~11 km upstream from the mouth of the Cochin estuary. The sclerochronological uncertainty of the record is about two weeks^18^. The δ^18^O and Δ_47_ measured across the growth bands of the clam shell capture a seasonal pattern (Fig. 2, Table 1). Rainfall in the region is prolonged due to the influence of two monsoon systems: the summer Southwest (SW) Monsoon from June to September, and the winter Northeast (NE) Monsoon from October to December. The δ^18^O of the water in the Cochin estuary close to the shell collection site ranged between −3.5‰_VSMOW_ during the entire period encompassing both the monsoons (June–December) and −0.5‰_VSMOW_ during the dry season (January–May)^19^. The corresponding carbonate growth bands had an average δ^18^O of −4.5‰_VPDB_ for the period of rain and −2.3‰_VPDB_ for the drier months. The record of daily rainfall at Cochin was obtained from the ‘World Weather’ TuTiempo Network (http://en.tutiempo.net) (Suppl. Information 2). It shows that rainfall, and hence freshwater input into the estuary, peaked in the period May through September, which corresponds to the period of thunderstorm activity during the pre-monsoon months (March–May) and increased rainfall during the monsoon (May–September) months.

**Table 1:** δ^13^C and δ^18^O values for *V. cyprinoides* shell growth bands and calculated δ^18^O of water reconstructed using observed monthly water temperature^45^.

Sample number	Assigned date	δ^18^O shell (‰VPDB)	δ^13^C shell (‰ VPDB)	δ^18^O water (‰ VSMOW)	Measured^+^temperature (°C)	Calculated^*^temperature (°C)
1	Dec-09	−3.87	−7.30	−1.58	30.1	29.55
2	Oct-09	−3.97	−8.91	−2.36	31.0	26.56
3	Sep-09	−4.31	−10.00	−2.01	29.4	29.59
4	Aug-09	−3.60	−9.38	−2.50	27.8	24.28
5	Jun-09	−2.54	−7.20	−3.46	29.4	15.33
6	May-09	−2.32	−6.20	−0.87	31.7	25.85
7	Mar-09	−2.66	−6.36	−0.58	30.5	28.62
8	Feb-09	−3.40	−7.20	−0.36	29.9	32.89
9	Dec-08	−4.18	−7.38	−1.58	30.1	30.92
10	Nov-08	−4.36	−8.40	−1.19	29.9	33.45
11	Sep-08	−4.29	−8.68	−2.01	29.4	29.50
12	Aug-08	−4.48	−10.09	−2.50	27.8	28.18
13	Jun-08	−4.23	−9.29	−3.46	29.4	22.82
14	May-08	−3.42	−8.04	−0.87	31.7	30.72
15	Apr-08	−3.95	−7.25	−0.75	31.0	33.58
16	Feb-08	−4.07	−6.17	−0.36	29.9	35.86
17	Jan-08	−3.30	−5.86	−1.10	27.1	29.17
18	Nov-07	−2.94	−5.68	−1.19	29.9	27.16
19	Oct-07	−3.67	−5.94	−2.36	31.0	25.23
20	Aug-07	−3.89	−6.46	−2.50	27.8	25.56

^+^Geetha *et al*., 2010 (ref.^45^).

*Grossman and Ku, 1986 (ref.^41^) modified by Dettman *et al*.,1999 (ref.^12^).

The analysis of CO_2_ clumped isotopes (CO_2_ isotopologues) in aragonite growth bands of modern *V. cyprinoides* and bio-calcite of Cretaceous *Phygraea (Phygrea) vesicularis* makes it possible to reconstruct seasonal ambient water temperatures with an uncertainty of ±2 °C^20,21^. Seasonal temperatures calculated from clumped isotope ratios (Δ_47_) measured on the aragonite growth increments in the *V. cyprinoides* shell ranged between 20.4 and 32.5 °C (Fig. 2), with a mean value of 28 °C. These temperatures are close to the directly-measured temperatures of the estuarine water (Suppl. Fig. S5). As seen in Suppl. Fig. S5, a poor correlation (R^2^ of 0.36, P = 0.04) is seen with the measured temperature. We suspect the factors like uncertainty in the determination of water temperature and the difference in the surface and bottom water temperature could have influenced the correlation. Monthly mean temperatures for the year 2009–2010 approached a minimum of 26 ± 1 °C during the winter months (December-February), whereas the regional maximum of 31 ± 1 °C was recorded during the summer months of March and April^22^ (Fig. 2, Table 2).

**Table 2:** Temperature and freshwater contribution to the Cochin estuary reconstructed using clumped isotope ratios and δ^18^O in seasonal growth bands of *V. cyprinoides*.

Sample ID	δ^13^C (‰,VPDB)	δ^18^O (‰,VPDB)	Δ_47_	Calculated temperature (°C)^#^	*δ^18^O water (‰,VSMOW)	Freshwater (%)
ESC1	−8.22	−4.34	0.69	29.6	−0.55	35
ESC2	−6.84	−3.49	0.69	30.1	0.41	13
ESC3	−5.84	−3.28	0.69	30.5	0.69	7
ESC4	−5.96	−3.46	0.70	26.7	−0.24	28
ESC5	−7.56	−4.41	0.68	31.9	−0.18	27
ESC6	−7.21	−3.93	0.71	24.8	−1.08	47
ESC7	−6.30	−3.87	0.68	32.5	0.50	11
ESC8	−5.49	−3.59	0.69	28.9	0.07	21
ESC9	−5.04	−3.39	0.73	20.4	−1.45	56
ESC10	−5.00	−3.47	0.69	30.0	0.40	14
ESC11	−7.07	−4.52	0.72	23.5	−1.94	67

^#^Dennis *et al*., 2011(ref.^22^).

*Grossman and Ku, 1986 (ref.^41^) modified by Dettman *et al*., 1999 (ref.^12^).

The δ^18^O of precipitated carbonate (mollusc shell) depends on both the isotopic composition and temperature of the water. Once the temperature has been determined independently by CO_2_ clumped isotopes (Suppl. Fig. S2), the δ^18^O of the water can be calculated. High rainfall during the monsoon months coincides with water δ^18^O values as low as −3.5‰_VSMOW_, whereas during non-monsoon months, when the water is more saline, δ^18^O approached −0.4‰_VSMOW_. This seasonal change in the δ^18^O of water in the Cochin estuary can be modelled as mixing between two components: rainwater runoff and seawater. The average δ^18^O of rainwater collected in the region in different seasons (−3.5‰_VSMOW_)^23,24^ is used as the rainwater end-member and the δ^18^O of the Arabian Sea (0.9‰_VSMOW_)^25^ is used for seawater.

### Cretaceous climate reconstruction using *Phygraea (Phygraea) vesicularis*

The concentrations of atmospheric greenhouse gases were significantly higher in the Cretaceous than they are today, therefore the global temperature was higher than it is now. The average equatorial sea surface temperature is estimated to have been ~31 °C^26^, compared to ~27 °C at present. This higher temperature is likely to have induced changes in the hydrological cycle. During the Early Maastrichtian, the Indian plate was positioned further south at mid-latitudes (~30°S) relative to its present position in the northern hemisphere. The tropical to sub-tropical climate favoured the formation of coal and limestone, and the higher concentrations of greenhouse gases led to enhanced terrestrial productivity^27^.

A fossilized shell of the mollusc *Phygraea (Phygraea) vesicularis*, with well-preserved distinguishable carbonate growth bands (Suppl. Fig. S6), was recovered from Late Cretaceous (Early Maastrichtian) shell-bearing strata near the village of Ottakoil, Tamil Nadu, India. Siliciclastic sediments (Ottakoil Formation) immediately above the fossiliferous carbonate layer and a conformable off-lap of much younger fluvial sand deposits (Kallamedu Formation) represent a regressive marine sequence. An overlying conglomerate bed has structures indicative of deposition in a shallow marine environment.

Oxygen isotope analysis of the carbonate in the growth bands of *P. vesicularis* revealed a record of seasonal changes in seawater temperature and salinity. The δ^18^O and Δ_47_ of the shell carbonate, and a Ce anomaly in the adjacent Kallankurichchi limestone are indicators of evaporative, anoxic environmental conditions at the time of deposition^28^.

**Figure 3:**
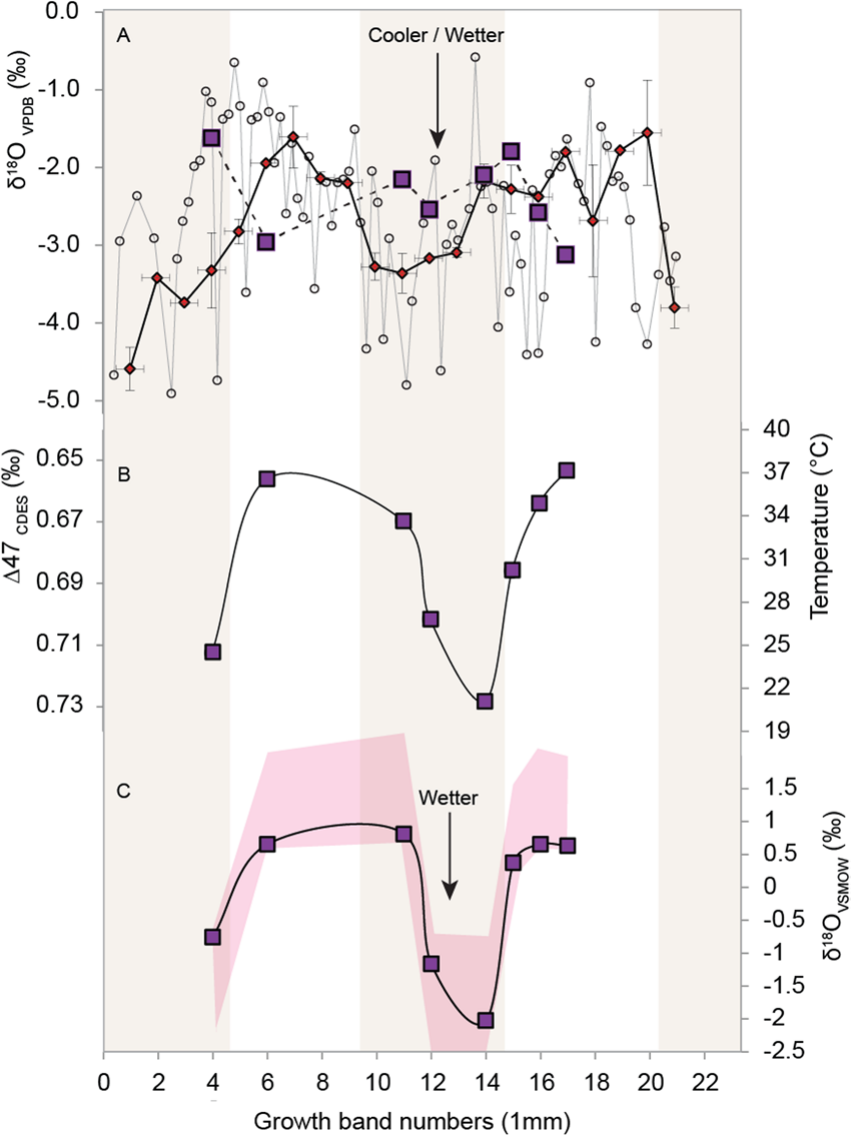
Reconstructed Early Maastrichtian temperature and seawater δ^18^O for the Cauvery Basin. (**A**) δ^18^O of *Phygraea vesicularis* shell 1 measured by dual inlet IRMS (squares) and shell 2 (diamonds) measured by continuous flow IRMS (1 mm resolution). Shell 2 was also analysed by SHRIMP with a 25 µm spot size and 150 µm spacing (circles). (**B**) Seasonal temperatures for shell 1 reconstructed using clumped isotope thermometry (CDES scale). (**C**) Calculated δ^18^O of water using IRMS shell δ^18^Odata in (**A**, **B**) and the empirical δ^18^O-temperature relationship for molluscan calcite of Dettman *et al*., 1999 (ref.^12^). Tan shading indicates cooler/wetter winter seasons.

The studied *P. vesicularis* shell had distinct prismatic and nacreous layers, no gross differences in Fe and Mn concentrations as measured by electron microprobe (Suppl. Information 3), and a lack of cathodoluminescence, indicating good preservation of its original composition^29,30^. Electron Backscattered Diffraction (EBSD) images of its growth structures, texture, crystal size and crystal orientation showed features similar to those characteristics of seasonal growth in modern day estuarine oysters (Suppl. information 1 Section S7, Figs S7, S8), consistent with there having been little or no diagenetic alteration^28^. The shell also had a large and systematic range of δ^18^O (>3‰, Fig. 3) as measured by both gas-source isotope ratio mass spectrometry (GIRMS) and Sensitive High Resolution Ion Micro Probe (SHRIMP).

The analysed section of shell recorded two cycles of δ^18^O, reflecting repetition of the seasonal change in temperature and salinity (Fig. 3). The temporal variation in δ^18^O followed a sinusoidal pattern, with higher δ^18^O in summer and lower δ^18^O in winter. The GIRMS measurements of aliquots of calcite micro-drilled from the shell showed a seasonal change in δ^18^O of ~3‰ (−1.5 to −4.6‰) with a mean value of −2.7‰ (n = 21). The individual SHRIMP microanalyses (n = 125) had a larger range (~6‰). The differences in δ^18^O between adjacent 25 µm spots were particularly large for the periods of summer growth (up to 3‰), much larger than can be explained by analytical uncertainty (±~0.3‰) or temperature fluctuations (Table 3). This variability coincided with changes in the crystal structure of the shell (Suppl. Fig. S6).The coarsely crystalline growth bands, mostly denoting summer growth, were more heterogeneous than the finely crystalline bands formed during winter. The Δ_47_ determined from the clumped isotope analyses ranged from 0.66 to 0.73‰, corresponding to a temperature range of 21 to 37 °C, with a mean value of 30 °C. This temperature range accounts for about 3.5‰ of the variation in the measured δ^18^O. All remaining variation (~2.5‰) is due to changes in the δ^18^O of the water in which the mollusc grew.

**Table 3:** Early Maastrichtian temperatures and freshwater inputs for the Cauvery Basin of southern India reconstructed using clumped isotope and δ^18^O analyses of seasonal growth bands in *P. vesicularis*.

Sample ID	Distance (mm)	δ^13^C (‰,VPDB)	δ^18^O (‰,VPDB)	Δ_47_ (Absolute Scale)	Calculated temperature (°C)^#^	δ^18^O* (‰,VSMOW)	Freshwater (%)
CRE A1	4	0.41	−1.62	0.71	24	1.6	17
CRE A2	6	0.61	−2.96	0.66	37	1.7	4
CRE A3	11	1.98	−2.15	0.67	34	1.6	1
CRE A4	12	1.12	−2.54	0.70	27	−0.5	22
CRE A5	14	1.07	−2.10	0.73	21	0.2	31
CRE A6	15	2.35	−1.79	0.69	30	1.9	5
CRE A7	16	1.66	−2.58	0.67	35	1.7	3
CRE A8	17	3.54	−3.12	0.66	37	0.7	5

^#^Dennis *et al*., 2011 (ref.^22^).

*Kim and O’Neil, 1997 (ref.^42^).

## Discussion

The well-preserved *P. vesicularis* shell recovered from Late Cretaceous sediments of the Cauvery Basin is an ideal specimen with which to determine the past rainfall seasonality pattern on the Indian coast when the subcontinent was positioned at latitude ~30°S. The observed variations in Cretaceous seawater δ^18^O and temperature are similar to those found in a modern coral-based proxy record from the Dampier Archipelago^27^, Western Australia (20.5°S), which shows the strong seasonal effect of evaporation on salinity. Coupled analyses of Sr/Ca and δ^18^O in the coral growth bands indicate that sea surface salinity during the period 1988–1994, as calculated from the residual δ^18^O after the temperature component was subtracted, was highest when strong evaporation in summer caused salinity (and water δ^18^O) and to increase. In contrast, reduced evaporation and increased rainfall in winter cause a decrease in seawater δ^18^O and salinity^27^.

The seasonal range in Early Maastrichtian water temperature in the Cauvery Basin was 21–37 °C, so the −1.6 to −3.1‰ range in δ^18^O measured in the *P. vesicularis* shell growth bands translates to a range in water δ^18^O from −0.5 to 1.7‰_VSMOW_ (Table 3). The seasonal change in freshwater contribution to the Cretaceous estuary can be calculated assuming the average δ^18^O of Early Maastrichtian rainfall to be about −6‰_VSMOW_ (inferred from the δ^18^O of contemporaneous palaeosol carbonates^7^) and seawater in the evaporating enclosed basin to have a δ^18^O of ~2‰ (as in the modern Red Sea—global seawater δ^18^O database; Craig, 1966). Based on this two end-member mixing model, the seasonal change in freshwater contribution to the Cretaceous estuary ranged from 3 to 31% between summer and winter (Table 3).

Further, water δ^18^O and temperature are strongly correlated, with the lowest temperatures associated with the lowest water δ^18^O values, suggesting a climatological control on the amount of runoff to the estuary. Temperature and the δ^18^O of precipitation are directly correlated with latitude^7^ whereby the δ^18^O of Late Cretaceous precipitation was lower while India was located in the southern hemisphere mid-latitudes. This conclusion is consistent with a Late Cretaceous palaeoclimate reconstruction from further south at Seymor Island^31^, where maximum freshwater discharge occurred during winter, suggesting warmer winters than those currently experienced in coastal regions in southern mid-latitudes.

The sedimentary record of the Kallankuruchchi Formation provides a further opportunity to probe the Late Cretaceous temperature and hydrological seasonality. Previous studies have interpreted sedimentary structures in the Kallankuruchchi Formation (cross bedding, cut and fill, hummocky cross stratification) as indicating deposition during major storm events. Evidence of storm deposition is also found in other contemporaneous strata in the Cauvery basin^32^. The weight of evidence is that, unlike the modern day, Cretaceous southern India experienced the bulk of its rainfall in winter, whereas reduced rainfall and runoff in summer increased both the salinity and temperature of the estuarine water, resulting in deposition of thick piles of evaporite and carbonate sediments in the stratigraphic succession.

The climatic conditions experienced in southern India at ~30°S latitude during the Late Cretaceous were evidently similar to those of the modern-day coastal plain of Western Australia around the same latitude, where seasonal storms and cyclones impact on the carbonate platform^33^. Our finding suggests that large-scale atmospheric circulation and seasonal hydroclimate patterns at mid-latitudes during the Cretaceous global warming interval were not substantially different from the present-day. The result is relevant for climate models designed to simulate the extent to which elevated atmospheric CO_2_ levels, and the accompanying global warming, might alter the Hadley circulation and mid-latitude storms in the future.

## Methods

### Background

The Cochin Backwaters at Kerala, India (9°40′ N–10°08′ N, 76°11′ E–76°25′ E) is populated by several species of mollusc, the most widespread being *Villorita*, known for its edible value. The area occupies the northern part of the Vembanad-Kol wetlands, and covers ~255 km^2^, extending from Alleppey to Cochin before merging with the Arabian Sea via two permanent openings. The region has a modern-day tropical climate with two main rainy seasons. Most of the rainfall (~70%) occurs during the SW monsoon from June to September. Much of the remaining rainfall (~15%) occurs during the NE monsoon from October to November, while the December-to-May pre-monsoon period has sporadic rainfall accounting for the remaining 15%. The surface water temperature in the estuary reaches a maximum of 32 °C in April and drops to as low as 24 °C in August.

### Sample collection

Several specimens of *Villorita cyprinoides* were harvested live from the southern extremity of the Cochin Backwaters on 20 January 2010 for the present investigation. The growth rate of *V. cyprinoides* varies over a year and is characterised by growth increments of aragonite separated by laminar bands. The average growth rate for the species is ~8.3 mm/year^16^, determined by monitoring a population of several individuals *in situ* within a cage experiment. For the present study, the incremental growth bands were drilled at a spatial resolution of 1 mm for δ^18^O and clumped isotope analysis.

The Cauvery Basin, at the southern tip of peninsular India (Fig. 1), hosts a complete Cretaceous sedimentary sequence of shallow marine to estuarine deposits. The sequence consists of the Uttatur, Trichinopoly and Ariyalur rock groups, representing Early, Middle and Late Cretaceous successions, respectively. Specimens of *Phygraea (Phygraea) vesicularis* were collected from an exposure in the Kallankuruchchi Formation (Ariyalur group), well exposed near the PNR mine of Dalmia Cement Limited (11°7′11′′N 79°7′59′′E)^34^.

### Recovery of carbonate from mollusc growth bands

In preparation for δ^18^Oand clumped isotope analysis, internal soft body parts were discarded from *V. cyprinoides* and the outer carbonate shells were treated with H_2_O_2_ for complete removal of organic debris, and then air dried for sectioning and drilling. Shells were dissected along the growth axis (Suppl. Figs S1a, S1b) using a section cutter and sampled along individual growth bands at 1 mm resolution using a battery operated micro mill. The recovered powder was analysed by XRD, showing aragonite to be the primary mineral phase.

The shell for the Cretaceous *P. vesicularis* was processed in a similar manner, sectioned along the growth axis and sampled at 1 mm and 2 mm resolution for δ^18^O and clumped isotope analysis. Powder analysed by XRD suggested calcite as the primary mineral in the Cretaceous specimen. The same section was polished for *in situ* analysis of δ^18^O at a spatial resolution of 25 μm by Sensitive High Resolution Ion Microprobe (SHRIMP).

### Conventional δ^18^O measurements

The δ^18^O (and δ^13^C) of the shell samples were measured at high-resolution using a Thermo Finnigan MAT 253 isotope ratio mass spectrometer (IRMS) coupled with a Gas bench II in continuous flow mode. About 100 μg of carbonate powder was reacted with 1 ml of H_3_PO_4_ using the boat method described elsewhere^35^ and the overall δ^18^O reproducibility for NBS-19 calcite was ±0.08‰. Water samples collected from the Cochin estuary were analysed for δ^18^O following the CO_2_-water equilibration method, where 100 μl of water was equilibrated with the CO_2_+He mixture for more than 18 hours (as described previously)^36^. The over-all δ^18^O reproducibility of replicate analyses of water standards was ±0.06‰.

Larger shell sample weights (~5–10 mg) required for the preparation of CO_2_ gas for clumped isotope measurements were obtained by combining powders from two or more growth bands (averaging ~2–3 months of growth). δ^18^O (and δ^13^C) in the larger samples were measured on the MAT 253 IRMS in dual inlet mode along with measurements of mass-47 isotopologues of CO_2_ for clumped isotope analysis. The overall δ^13^C, δ^18^O and Δ47 reproducibility for NBS-19 calcite value of ±0.04, 0.05 and 0.01‰ respectively.

### Measurements of Δ_47_ in shell growth bands

Δ_47_ analyses were performed using the dual inlet peripherals on the Thermo MAT 253 IRMS following the preparation steps of CO_2_ cleaning through use of an external GC setup^37^. The experimental procedure for sample preparation for clumped isotope analysis used the sealed vessel method of  ^38,39^. All carbonate samples were prepared in the experimental setup designed at the Indian Institute of Science, Bangalore. For each analysis, ~5–10 mg of carbonate powder was reacted with 1 ml of H_3_PO_4_ in a sealed reaction vessel. The reaction vessel was evacuated on a gas-extraction line to a pressure of 10^−4^ mbar using a combination of turbomolecular and roughing pumps. The stopcock in the evacuated vessel was then closed and the vessel kept in a water bath maintained at a constant temperature of 25 ± 0.1 °C. The reaction of carbonate with H_3_PO_4_ commenced by a simple transfer of acid from the arm of the reaction vessel to the compartment with carbonate powder.

The CO_2_ generated during the reaction was cleaned using a cryogenic extraction protocol to remove contaminants responsible for isobaric interferences^37^. Purification steps involved removal of water vapour and other contaminants by a combination of liquid nitrogen trap and a dry ice and ethanol slush trap. The CO_2_, once extracted onto a cold finger, was entrained with a He stream through a capillary column (PoraPLOT Q, 25 m × 0.32 mm i.d.; Varian Inc., Palo Alto, CA, USA) and held at −10 °C for gas chromatographic separation of CO_2_ from other mixtures of trace hydrocarbon and halocarbon. Eventually, the purified CO_2_ sample was taken into a glass cold finger and analysed using the MAT 253 IRMS dual inlet system.

The MAT 253 IRMS was configured to analyse mass-47 isotopologues of CO_2_ by simultaneously measuring mass 47, 48 and 49 (measured with 10^12^ Ω resistors). Masses 48 and 49 were monitored in order to ensure that there were no isobaric interferences due to the presence of contaminants. These measurements were done in dual inlet mode with a source pressure sufficient to maintain the CO_2_ mass-44 ion beam intensity at a voltage of 10–12 V. Each analysis involved 60 measurement cycles of the sample CO_2_ and reference CO_2_ (six acquisition lines with 10 cycles each, with a signal integration time of 8 s per measurement). The reference CO_2_ gas (Linde CO_2_) had δ^13^C and δ^18^O values of −4.41‰ (VPDB) and 24.59‰ (VSMOW), based on repeat analyses of the NBS-19 carbonate standard.

The Δ_47_ analyses were standardized using in-run measurements of NBS-19, heated gas and in-house MAR J1 calcite (generated from Carrara marble)^39^. Calibration of MARJ1, which was run more frequently during the course of our measurements, was done by adopting two published values for the other two reference materials (NBS-19 and heated CO_2_ at 1000 °C). The Δ_47_ value of 0.392‰ for NBS-19, and 0.026‰ for heated CO_2_, were adopted to relate the heated gas scale to the CDES scale^22^. The MAR J1 Carrara marble was assigned a value of 0.395‰ for scale conversion purposes. The long-term reproducibility of MAR J1 over the period 2010–2012 (n = 59) yielded a Δ_47_ value of 0.343 ± 0.01 (1σ) on the heated gas scale. All the shell carbonate samples were analysed during that period.

In order to convert the Δ_47_ values to the heated gas scale, a large number of CO_2_ samples were treated at 1000 °C for 2 hours upon recovery of the analysed samples. The CO_2_ samples used for high temperature treatment were obtained upon transferring the sample CO_2_ to an ultrapure synthetic quartz tube (6 mm o.d.), which was evacuated and sealed. The sealed tube containing the CO_2_ sample was heated in a muffle furnace at 1000 °C for >2 hours and then quickly quenched to room temperature. The difference between the Δ_47_ values measured in the sample CO_2_ and randomized CO_2_ generated upon heating at 1000 °C allowed the definition of the Δ_47-HG_ value in the heated gas scale^20^. All data are reported at the 25 °C reaction temperature and therefore no additional correction for the reaction temperature fractionation factor was applied. For the period of analysis, heated CO_2_ yielded Δ_47_ values (n = 66) of −1.47 ± 0.06‰ (1σ). The values on the heated gas scale are converted to the absolute CDES scale by using the proposed equation^22^ relating the absolute value of Carrara marble and heated gas and is given here as:

1 $$\begin{array}{c}{{\rm{\Delta }}}_{47}\,{\rm{sample}}\_{\rm{Abs}}=((({{\rm{\Delta }}}_{47{\rm{SAMPLE}}\_{\rm{HG}}}+1)\cdot ({{\rm{\Delta }}}_{47{\rm{MARJ}}1\_{\rm{HG}}}+1)/({{\rm{\Delta }}}_{47{\rm{MARJ}}1\_{\rm{WG}}}+1)\,\cdot \,\\ \,\,\,\,\,\,\,\,({\rm{\Delta }}47{\rm{HG}}\_{\rm{WG}}+1))-1)\cdot 1000\end{array}$$

where the subscripts ‘NBS_HG’, ‘NBS_WG’ and ‘HG_WG’ represent, respectively, the Δ_47_ value of the NBS-19 with respect to heated CO_2_, the NBS-19 value with respect to our Linde reference CO_2_ used here as working gas and the heated CO_2_ value with respect to Linde reference CO_2_ (working gas). The values for δ^18^O (and δ^13^C) in the carbonate samples were reported on the VPDB scale, while Δ_47_ values were expressed following the carbon dioxide equilibrium scale (CDES) scale^22^.

For deriving the δ^18^O of water from measurements of δ^18^O in carbonates, the carbonate δ^18^O values were converted to the VSMOW scale using the following equation^40^:

2 $${\delta }^{18}{\rm{O}}\,{\rm{V}}{\rm{S}}{\rm{M}}{\rm{O}}{\rm{W}}=1.03091\ast {\delta }^{18}{\rm{O}}\,{\rm{V}}{\rm{P}}{\rm{D}}{\rm{B}}+30.91$$

Furthermore, the water δ^18^O values for modern *V. cyprinoides* samples were deduced using the growth band δ^18^O, Δ_47_-based temperature and the relationship proposed for shell aragonite^41^:

3 $$T^\circ C=20.6-4.34({\delta }^{18}O\,(calcite)-{\delta }^{18}O\,(water))$$

where δ^18^O of aragonite is in VPDB and δ^18^O water is in VSMOW. Similarly, for Late Cretaceous *P. vesicularis* samples, water δ^18^O was deduced using the relationship for calcite^42^:

4 $$1000\,ln\,\alpha (calcite-{H}_{2}O)=18.03\times ({10}^{3}{T}^{-1})-32.42)$$

where the calcite-water fractionation factor (α) is defined as:

5 $$\alpha \,(calcite-{H}_{2}O)=(1000+{\delta }^{18}O\,(calcite))/(1000+{\delta }^{18}O\,(water))$$

### SHRIMP δ^18^O measurements

A glass-mounted polished thin section of the *Phygraea vesicularis* shell was cut to size and cast with grains of NBS-18 and NBS-19 reference calcite in a disc of Struers Epofix epoxy resin 35 mm diameter. The disc was lightly polished with 3 µm and 1 µm diamond paste to expose the standards, degreased, photographed at high magnification, washed with petroleum spirit, warm detergent solution and Millipore water, dried in a 60 °C vacuum oven for 24 hours and coated with 12 nm of high purity Al before being loaded into the Australian National University SHRIMP II for O isotopic analysis.

The analytical procedure was based on that described by Ickert *et al*.^43^ and Long *et al*.^44^. In brief, a ~3 nA primary ion beam of ~15 kV Cs^+^ was focused to a probe ~25 µm in diameter, and secondary ions of O^−^(^16^O ≈ 1.9 × 10^9^ c/s) were extracted at ~10 kV for isotopic analysis by dual Faraday cup multiple collection (current mode, 10^11^ Ω resistors). Charge build-up on the sample surface was neutralised using a focused ~600 eV electron beam. Each analysis consisted of a 90 s pre-burn during which electrometer baselines were measured, ~2 min of ion focusing and 12 × 10 s measurements of ^18^O/^16^O. Internal precision of each spot analysis was ≤0.15‰ (s.e.). Corrections for electron induced secondary ion emission (EISIE) were ~0.1‰. δ^18^O was calculated relative to analyses of several fragments of NBS-19 (δ^18^O_VPDB_ = −2.2‰) distributed throughout the 20-hour analytical session. Reproducibility of the analyses of NBS-19 over the whole session was 0.21‰ (s.d., n = 27).

## Electronic supplementary material

Supplementary information 1

Supplementary information 2

Supplementary information 3

## References

[1] Feng X, Porporato A, Rodriguez-Iturbe I (2013). Changes in rainfall seasonality in the tropics. Nat. Clim. Change.

[2] Pachauri, R. K. *et al*. Climate change 2014: synthesis report. Contribution of Working Groups I, II and III to the fifth assessment report of the Intergovernmental Panel on Climate Change. (IPCC, 2014).

[3] Barclay RS, Wing SL (2016). Improving the Ginkgo CO_2_ barometer: Implications for the early Cenozoic atmosphere. Earth and Planetary Science Letters..

[4] Miller KG, Barrera E, Olsson RK, Sugarman PJ, Savin SM (1999). Does ice drive early Maastrichtian eustasy?. Geology..

[5] Hasegawa H (2012). Drastic shrinking of the Hadley circulation during the mid-Cretaceous Super Greenhouse. Climate of the Past..

[6] Floegel S, Wagner T (2006). Insolation-control on the Late Cretaceous hydrological cycle and tropical African climate—global climate modelling linked to marine climate records. Palaeogeography, Palaeoclimatology, Palaeoecology..

[7] Ghosh, P. *et al*. Tracking the migration of the Indian continent using the carbonate clumped isotope technique on Phanerozoic soil carbonates Scientific Reports. **6** (2016). 10.1038/srep22187 PMC4773985 26931069

[8] Sastri V, Venkatachala B, Narayanan V (1981). The evolution of the east coast of India. Palaeogeography, Palaeoclimatology, Palaeoecology..

[9] Chari MVN, Sahu JN, Banerjee B, Zutshi PL, Chandra K (1995). Evolution of the Cauvery Basin, India from subsidence modeling. Marine and Petroleum Geology..

[10] Rao LR (1956). Recent contributions to our knowledge of the cretaceous rocks of South India. Proceedings of the Indian Academy of Sciences-Section B.

[11] Madhavaraju J (2015). Carbon, oxygen and strontium isotopic signatures in Maastrichtian-Danian limestones of the Cauvery Basin, South India. Geosciences Journal..

[12] Dettman DL, Reische AK, Lohmann KC (1999). Controls on the stable isotope composition of seasonal growth bands in aragonitic fresh-water bivalves (unionidae). Geochimica et Cosmochimica Acta..

[13] Banerjee, Y., Ghosh, P., Bhushan, R. & Rahul, P., Strong sea forcing and warmer winter during solar minima ~2765 yr BP recorded in the growth bands of Crassostrea sp. from the confluence of river Ganges, Eastern India., Quaternary International. in press (2017).

[14] Jacob B, Revichandran C, Kumar N (2013). Salt intrusion study in Cochin estuary-using empirical models. Indian Jour. Of Marine Science..

[15] Vinita J (2015). Salinity response to seasonal runoff in a complex estuarine system (Cochin Estuary, west coast of India). Journal of Coastal Research..

[16] Arun, A. U. Gametogenic cycle in Villorita cyprinoides and the influence of salinity. Aquaculture, Aquarium, Conservation & Legislation-International Journal of the Bioflux Society (AACL Bioflux). **2** (2009).

[17] Arun AU (2009). An assessment on the influence of salinity in the growth of black clam (Villorita cyprinoides) in cage in Cochin Estuary with a special emphasis on the impact of Thanneermukkom salinity barrier. Aquaculture, Aquarium, Conservation & Legislation-International. Journal of the Bioflux Society (AACL Bioflux)..

[18] Schone BR, Tanabe K, Dettman DL, Sato S (2003). Environmental controls on shell growth rates and delta O-18 of the shallow-marine bivalve mollusk Phacosoma japonicum in Japan. Marine Biology..

[19] Kaushal R, Ghosh P, Geilmann H (2016). Fingerprinting environmental conditions and related stress using stable isotopic composition of rice (Oryza sativa L.) grain organic matter. Ecological Indicators..

[20] Eiler JM, Schauble E (2004). ^18^O ^13^C ^16^O in Earth’s atmosphere. Geochimica et Cosmochimica Acta..

[21] Schauble EA, Ghosh P, Eiler JM (2006). Preferential formation of C-13-O-18 bonds in carbonate minerals, estimated using first-principles lattice dynamics. Geochimica et Cosmochimica Acta..

[22] Dennis KJ, Affek HP, Passey BH, Schrag DP, Eiler JM (2011). Defining an absolute reference frame for ‘clumped’ isotope studies of CO_2_. Geochimica et Cosmochimica Acta..

[23] Unnikrishnan AS, Shankar D (2007). Are sea-level-rise trends along the coasts of the north Indian Ocean consistent with global estimates?. Global and Planetary Change..

[24] Lekshmy, P. R., Midhun, M., Ramesh, R. & Jani, R. A. O-18 depletion in monsoon rain relates to large scale organized convection rather than the amount of rainfall. Scientific Reports. **4** (2014). 10.1038/srep05661 PMC4092346 25012535

[25] Singh A, Jani RA, Ramesh R (2010). Spatiotemporal variations of the delta O-18-salinity relation in the northern Indian Ocean. Deep-Sea Research Part I-Oceanographic Research Papers..

[26] Zakharov YD (2011). Cretaceous climate oscillations in the southern palaeolatitudes: New stable isotope evidence from India and Madagascar. Cretaceous Research..

[27] Gagan MK (2000). New views of tropical paleoclimates from corals. Quaternary Science Reviews..

[28] Nagendra, R., Sathiyamoorthy, P. & Reddy, A. N. In STRATI 2013: First International Congress on Stratigraphy At the Cutting Edge of Stratigraphy (eds Rogério Rocha, João Pais, Carlos José Kullberg, & Stanley Finney) 547–551 (Springer International Publishing, 2014).

[29] Jacob DE (2008). Nanostructure, composition and mechanisms of bivalve shell growth. Geochimica et Cosmochimica Acta..

[30] Pérez-Huerta A, Cuif JP, Dauphin Y, Cusack M (2014). Crystallography of calcite in pearls. European Journal of Mineralogy.

[31] Petersen, S. V., Dutton, A. & Lohmann, K. C. End-Cretaceous extinction in Antarctica linked to both Deccan volcanism and meteorite impact via climate change. Nature Communications **7** (2016). 10.1038/ncomms12079 PMC4935969 27377632

[32] Ramkumar MA (2006). storm event during the Maastrichtian in the Cauvery basin, south India. Geoloski anali Balkanskoga poluostrva.

[33] Berry P, Marsh L (1986). History of investigation and description of the physical environment. Faunal Surveys of The Rowley Shoals, Scott Reef and Seringapatam Reef North-West Australia: Records of the Western Australian Museum, Supplement.

[34] Ayyasami K (2006). Role of oysters in biostratigraphy: A case study from the Cretaceous of the Ariyalur area, southern India. Geosciences Journal..

[35] Rangarajan, R. High Resolution Reconstruction of Rainfall Using Stable Isotopes in Growth Bands of Terrestrial Gastropod Doctor of Philosophy (Ph.D.) thesis, Indian Institute of Science (2014).

[36] Rangarajan R, Ghosh P (2011). Role of water contamination within the GC column of a GasBench II peripheral on the reproducibility of O-18/O-16 ratios in water samples. Isotopes in Environmental and Health Studies..

[37] Ghosh P (2006). (13)C-(18)O bonds in carbonate minerals: A new kind of paleothermometer. Geochimica et Cosmochimica Acta..

[38] McCrea JM (1950). On the isotopic chemistry of carbonates and a paleotemperature scale. The Journal of Chemical Physics.

[39] Rangarajan R, Ghosh P, Naggs F (2013). Seasonal variability of rainfall recorded in growth bands of the giant African land snail Lissachatina fulica (Bowdich) from India. Chemical Geology..

[40] Coplen TB, Kendall C, Hopple J (1983). Comparison of stable isotope reference samples. Nature.

[41] Grossman EL, Ku TL (1986). Oxygen and carbon isotope fractionation in biogenic aragonite: temperature effects. Chemical Geology (Isotope Geoscience Section).

[42] Kim ST, O’Neil JR (1997). Equilibrium and nonequilibrium oxygen isotope effects in synthetic carbonates. Geochimica et Cosmochimica Acta..

[43] Ickert RB (2008). Determining high precision, *in situ*, oxygen isotope ratios with a SHRIMP II: Analyses of MPI-DING silicate-glass reference materials and zircon from contrasting granites. Chemical Geology..

[44] Long K (2014). Fish otolith geochemistry, environmental conditions and human occupation at Lake Mungo, Australia. Quaternary Science Reviews..

[45] Geetha, P., Thasneem, P. & Nandan, S., Macrobenthos and Its Relation to Ecosystem Dynamics in the Cochin Estuary, in Lake 2010: Wetlands, Biodiversity and Climate Changes (IISc, Bangalore, 2010), 1–12 (2010).

