# Author Correction: Rainfall seasonality on the Indian subcontinent during the Cretaceous greenhouse

**DOI:** 10.1038/s41598-018-28639-9

**Published:** 2018-07-03

**Authors:** Prosenjit Ghosh, K. Prasanna, Yogaraj Banerjee, Ian S. Williams, Michael K. Gagan, Atanu Chaudhuri, Satyam Suwas

**Affiliations:** 1 Centre for Earth Sciences, Indian Institute of Science, Bangalore, 560012 India; 2 Divecha Centre for Climate Change, Indian Institute of Science, Bangalore, 560012 India; 3 Birbal Sahni Institute of Palaeosciences, 53, University Road, Lucknow, 226007 India; 4 Research School of Earth Sciences, The Australian National University, Acton, ACT 2601 Australia; 5 School of Earth and Environmental Sciences, The University of Queensland, Brisbane, QLD 4072 Australia; 6 Department of Materials Engineering, Indian Institute of Science, Bangalore, 560012 India

Correction to: *Scientific Reports* 10.1038/s41598-018-26272-0, published online 31 May 2018

The Acknowledgements section in this Article was omitted. The Acknowledgements section should read:

“P.G thanks Department of Science and Technology, Govt. of India (SR/S4/FS-481/2009), Ministry of Earth Sciences (MoES/ATMOS/PP-IX/09), Divecha Centre for Climate Change, Australian Scientific Instrument and I.W thanks Australian Scientific Instrument for funding the project.”

