# Supplementary material for: Rainfall seasonality on the Indian subcontinent during the Cretaceous greenhouse
Source: Sci Rep. 2018 May 31;8:8482. doi: 10.1038/s41598-018-26272-0 (PMC5981374; doi:10.1038/s41598-018-26272-0)
Supplement: Supplementary file 1 — Supplementary information 1 [file 41598_2018_26272_MOESM1_ESM.docx]

**Supplementary Information**

**Rainfall seasonality on the Indian subcontinent during the Cretaceous Greenhouse**

Prosenjit Ghosh^1, 2*^, K. Prasanna^1, 3^, Yogaraj Banerjee^1^, Ian S. Williams^4^,

Michael K. Gagan^4,5^, Atanu Chaudhuri^6^, Satyam Suwas^6^

1 Centre for Earth Sciences, Indian Institute of Science, Bangalore, 560012, India

2 Divecha Centre for Climate Change, Indian Institute of Science, Bangalore, 560012, India

3 Birbal Sahni Institute of Palaeosciences, 53, University Road, Lucknow, 226007, India

4 Research School of Earth Sciences, The Australian National University, Acton, ACT, 2601,Australia

5 School of Earth and Environmental Sciences, The University of Queensland, Brisbane, QLD, 4072, Australia

6 Department of Materials Engineering, Indian Institute of Science, Bangalore, 560012, India

*Corresponding author: ghoshceas@gmail.com

**S1. Nature of samples used in this investigation**

Several samples of *V. cyprinoides* were collected from the Cochin backwater during January 2010 for the present study. The regional water body where these shells grew is fed by rainwater and river discharge. A barrage was commissioned in the year 1976 to regulate the flow of seawater to the estuary. The growth bands present within the mollusc shell showed distinct coloration, dark and light markings representing winter and summer time precipitates. XRD analysis of the shell powder showed 2θ peaks for the carbonate mineral aragonite. The occurrence of *V. cyprinoides* is restricted to the zone where salinity is low, and it has been suggested that their growth rate varies inversely with salinity^12,13^. *V. cyprinoides* in the Cochin backwater have been widely studied for their growth rate, where seasonal variability of the freshwater supply causes shifts in salinity and nutrient level, affecting growth patterns. A range of values from 8.3 to 11.2 mm/yr ^12,13^has been reported based on observations.

**
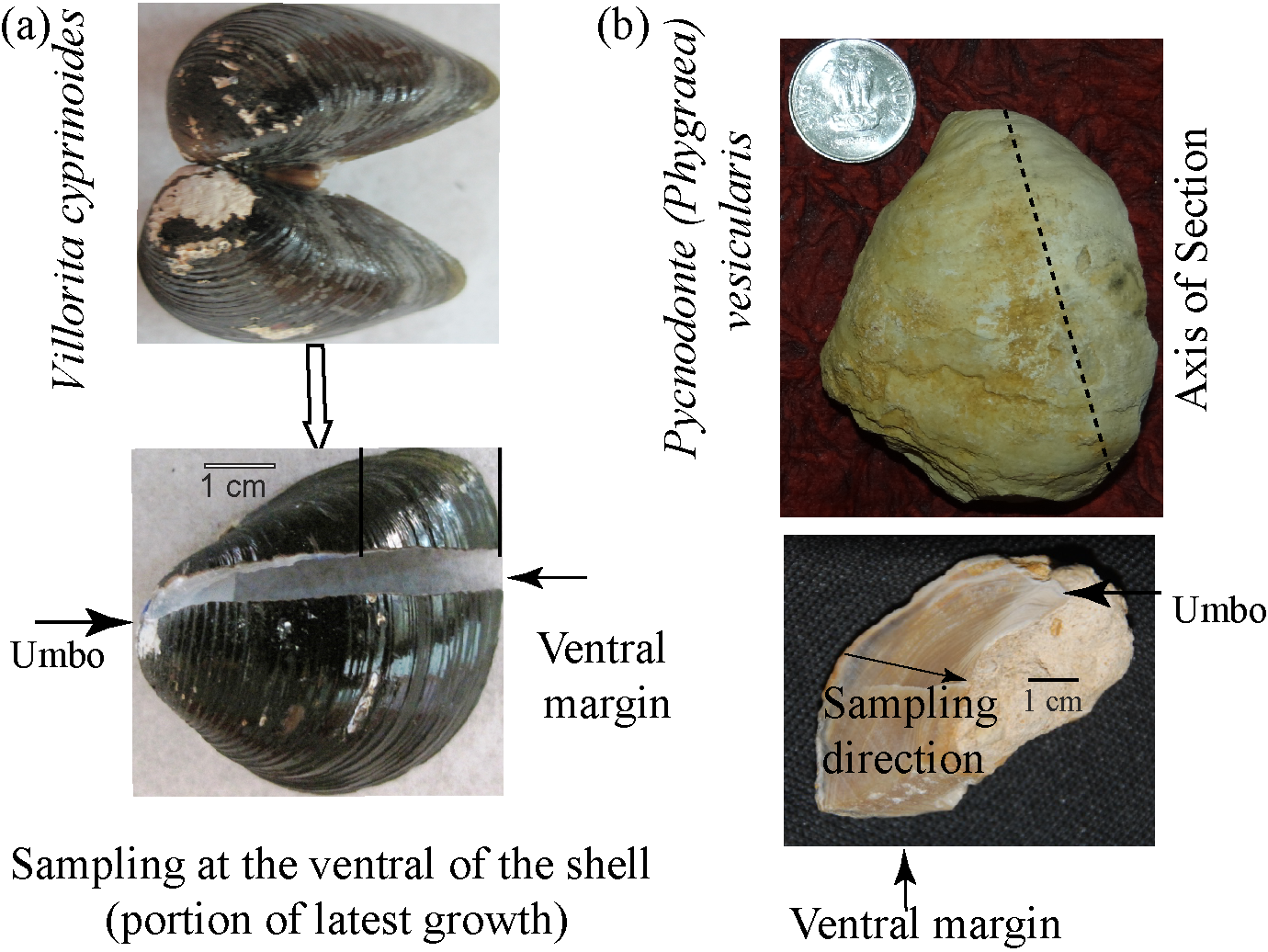
**

Figure S1: (a) Image of a live sample of *Villorita cyproniods* showing distinct bands with colouration (like tree rings) that were added to the shell as it grew in size. The isotopic signature of the bands mimics the environmental conditions (temperature and salinity) in which the organism lived. This means that the shell’s growth bands preserve an isotopic pattern that reflects their habitat and growth condition. (b) A section image of *Phygraea vesicularis* displaying growth lines with well-preserved structure. XRD analysis of the shell powder showed 2θ peaks for the carbonate mineral calcite. Incremental growth bands were sampled using a microdrill for conventional and clumped isotopic analysis.

**S2. Reversal of seasonality based on temperature and water isotopes**

Figure S2 shows the correlation (R^2^ = 0.73, p<0.001) between temperature and δ^18^O in modern *V. cyproniods* shell samples. We interpret this correlation as a seasonal signal reflecting the variable contribution of freshwater from continental runoff at seasonal time intervals. Rainfall and river discharge contribute most to the overall freshwater input to the estuary during monsoon time, while drier periods witness more seawater intrusion. The observed δ^18^O variability in the estuarine water reflects the influence of high discharge during summer time.


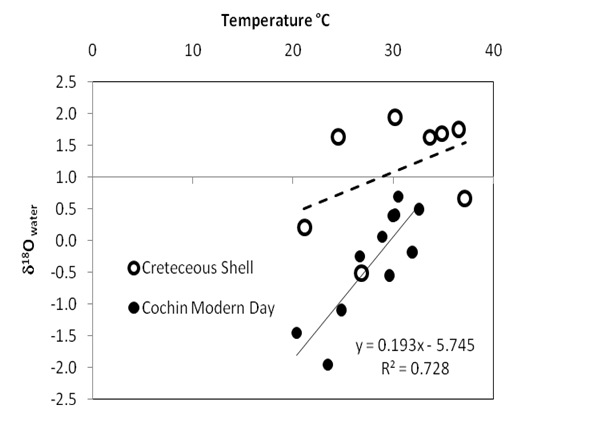


Figure S2: Relatively low δ^18^O values in modern *V. cyproniods* occur during cooler periods mainly coinciding with high runoff into the estuary. The correlation between δ^18^O and temperature is strong suggesting a climatological control on runoff delivery. Precipitation becomes more depleted in ^18^O as air temperature reduces due to consistent cloud cover during the active monsoon time.

During the Cretaceous, when the Indian plate was positioned at ~30**°**S, the temperature and δ^18^O relationship given by analysis of *Phygraea vesicularis shell material* was similar to the present-day with storm occurrences during winter allowing freshwater accumulation in near shore water bodies. This interpretation is consistent with observations documented in modern-day fringing reef corals from Western Australia^30^.

**S3. Nature of preservation of the modern shell**

Figure S3 shows a thick section and backscattered image of a modern *Villorita cyproniods* shell from the Cochin estuary. Note the clear seasonal growth bands.


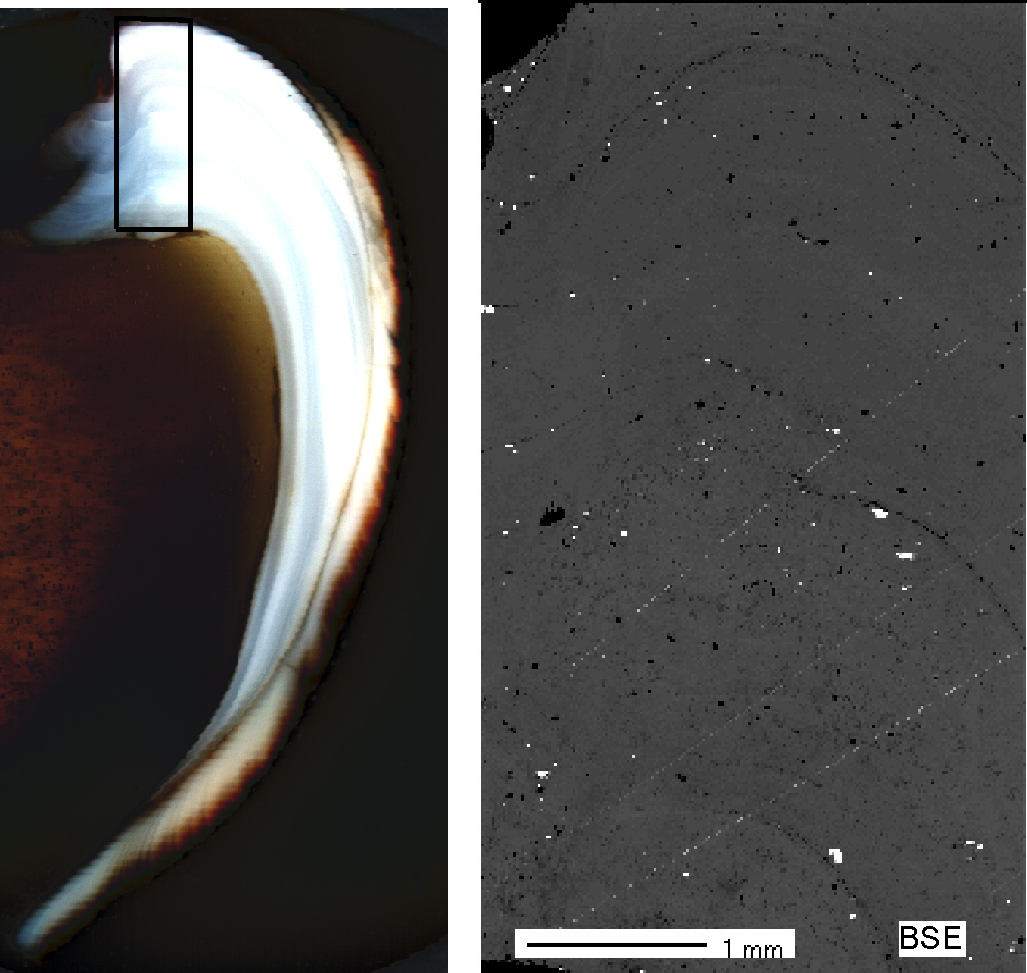


FigureS3: Section of modern shell from theCochin estuarywith laminar growth bands, which were micro-drilled at 1 mm and 5mmresolution for conventional stable isotope analysis and clumped isotope analysis, respectively.

**S4. Relationship between rainfall and freshwater contribution to the Cochin estuary**

The calculated freshwater contribution to the Cochin estuary, based on the mixing model described in the text with seawaterδ^18^O (0.9‰_SMOW_) and rainwaterδ^18^O (-3.5‰_SMOW_) end-members, is correlated with cumulative river discharge data for the Periyar River. We used mean monthly data for the Periyar River, draining into the estuary for the period of our observations (Table S1), to obtain the cumulative river discharge values (Table S2).

Supplementary Table S1. Mean monthly discharge data for the Periyar River (contributing freshwater to the Cochin estuary) for the 2008-2009 year.

| Month | Mean (m^3^/sec) |
| --- | --- |
| Nov | 160 |
| Dec | 150 |
| Jan | 90 |
| Feb | 90 |
| Mar | 20 |
| Apr | 30 |
| May | 70 |
| Jun | 190 |
| Jul | 250 |
| Aug | 420 |
| Sep | 360 |
| Oct | 250 |

Supplementary Table S2. Cumulative discharge data for the Periyar River corresponding to the date assigned to shell growth bands (see Table 1 in text). These data were obtained from monthly mean values given in Jacob et al., 2013 (ref. 14) for the 2008-09 year.

| Date | Cumulative river discharge 2008-09 (m^3^/sec) |
| --- | --- |
| Dec-09 | (Dec, Jan) 120 |
| Jun-09 | (Nov-May) 87 |
| May-09 | (May,June)130 |
| Mar-09 | (Dec-Apr) 76 |
| Nov-08 | (Nov-Feb) 123 |
| May-08 | (May-Oct) 257 |
| Apr-08 | (Apr, May) 50 |
| Feb-08 | (Jan-May) 60 |
| Jan-08 | (Nov-Jan) 133 |
| Nov-07 | (Nov, Dec) 155 |
| Aug-07 | (Aug-Oct) 343 |

The cumulative river discharge data for the Periyar River was plotted against estimates of freshwater contribution (in percent) to show the efficacy of our mixing model for the Cochin estuary (Fig. S4). This shows that the clumped isotope ratios and δ^18^O of shell growth bands are suitable recorders of salinity conditions and are thus useful for the reconstruction of river discharge back through geological time. We used this approach to reconstruct Late Cretaceous seasonal freshwater discharge.


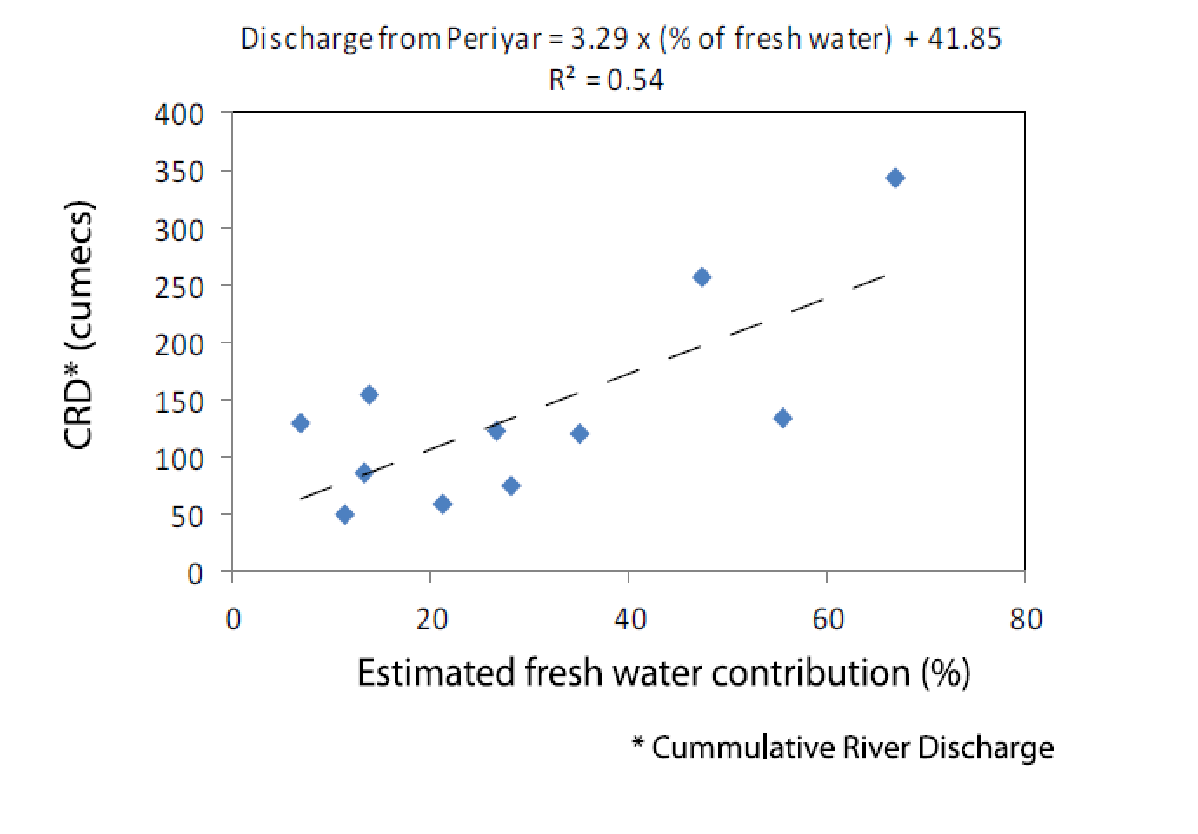


Figure S4: This plot displays the strong relationship (R^2^=0.54, p<0.0001) between percentage freshwater contribution and cumulative river discharge (CRD) of the Periyar River, the major river draining into the Cochin estuary.

**S5. Measured temperature and clumped isotope temperature estimates**

The clumped isotope based temperature estimates showed a strong correlation (R^2^ = 0.36, p=0.0484) with temperatures measured in the Cochin estuary (Fig. S5). The measured temperatures match closely with clumped isotope based estimates for the warmer times. However, the differences between the temperature estimates and measured temperatures increase for analyses of shell carbonate bands deposited during winter.


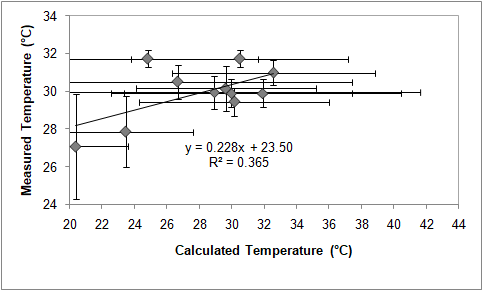


Figure S5: Relationship between measured temperatures and temperatures calculated using clumped isotope thermometry.

**S6. Nature of preservation of the Cretaceous shell**

The shell microstructure for the *Phygraea* vesicularis specimen analysed in this study showed primary features that are distinguishable based on crystal orientation and crystal arrangements seen in backscattered electron (BSE) images. Repetition of prismatic structure and lamellar structure is suggestive of differential growth conditions. A transitional zone between the two structures (up to 10 μm) is filled with acicular prisms, with longitudinal axes generally perpendicular to the depositional*.* The changes in the thickness of shell microstructures were synchronized with seasonal changes in water composition. Isotopic investigation using the ANU Sensitive High Resolution Ion Microprobe (SHRIMP) revealed that the crossed lamellar structure thickening occured at higher temperatures during summer, whereas prismatic layers were deposited during winter.


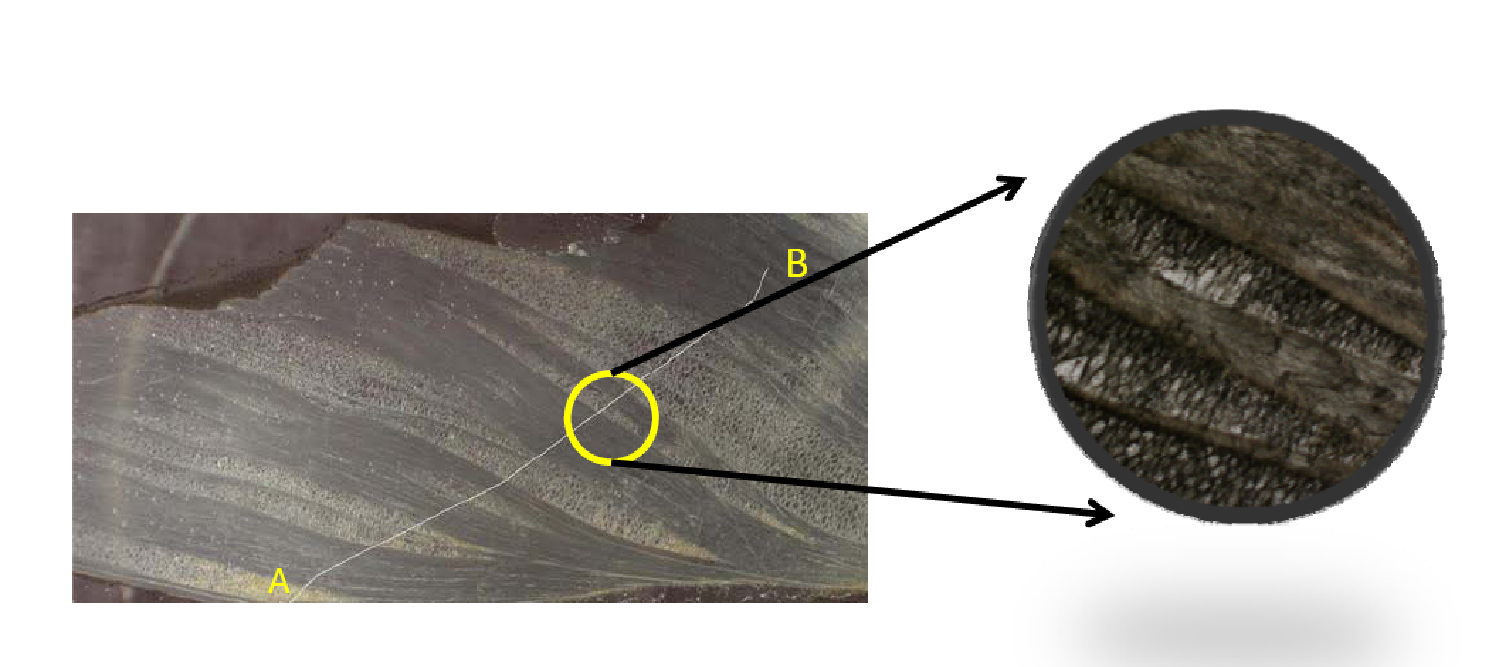


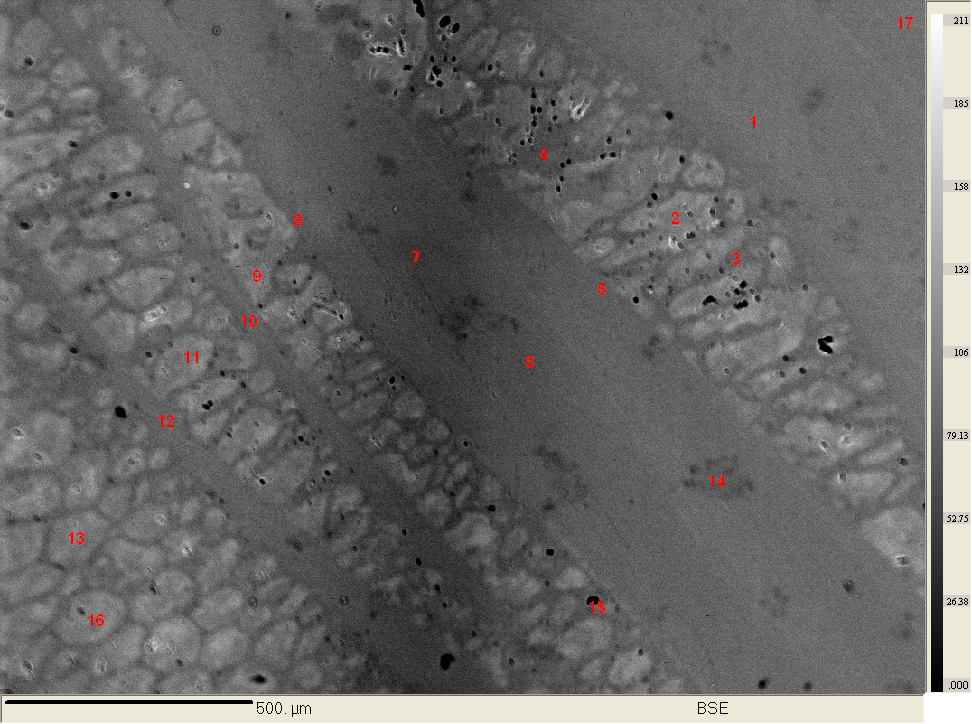


Figure S6: Thin section of the Cretaceous *Phygraea* vesicularis specimen showing preservation of alternate laminar and prismatic bands. Micro-drilled powder samples were retrieved for convention alanalysis of δ^18^Ousing a MAT 253 IRMS and Gas Bench II.Clumped isotope measurements were obtained using the dual inlet ofthe MAT 253. The combination ofδ^18^Odata and clumped isotope temperature estimates allowed retrieval of the waterδ^18^O values during the period of shell growth. The yellow line denoted as AB shows the sampling track chosen for SHRIMP (see data in Fig. 3 of paper). The circular area (yellow)shows the close-up view of crystal orientation(on right), which was further studied using backscattered electron (BSE) and electron backscattered diffraction (EBSD)imaging to assess shell preservation potential.

**S7.Electron backscattered diffraction (EBSD) study**

We used electron backscattered diffraction (EBSD) in the scanning electron microscope (SEM) to study the degree of change in the orientation of shell microstructure and crystal grain. Modern shells similar in kind to ours were investigated in a separate study by Huerta et al. (2014)(ref. 46)*.* Here we compare the results for both studies to show the crystal orientation. To be consistent, we transformed the coordinate system with a rotation of 90° to compare with the modern example, as shown in Figure S7.


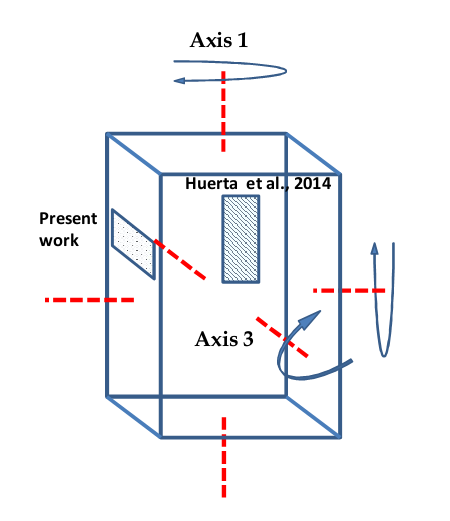


Figure S7: Schematic representation showing positional difference between the planes on which EBSD analysis was carried out for this study (marked by the white rectangle) and for the study of Huerta et al., 2014 (marked by the blue rectangle). For the purpose of comparison, we have corrected/reoriented our data with a 90°rotationalong Axis 1 followed by another 90° rotation along Axis 3, and have shown inthe pole figures in Fig. S8 (D,E,F)below. All the isotopic measurements in our study were carried out on the same plane as the EBSD analysis.

The sample section showing the EBSD micrograph of the Cretaceous *Phygraea* vesicularis shell is shown in Figure S8and compared with the EBSD micrograph of shell material analysed by Huerta et al. (2011, 2014) in panels B and C. The SEM image of the analysed area was converted into a pole figure and orientation map. Analysis of the orientation of individual grains is plotted in the pole figures, which shows a fair degree of consistency with each other (see panels D, E and F in Fig. S8). We used this as an index to argue in favour of no or minimal diagenetic transformation of the original isotopic signature. The crystal orientation displayed in the pole figures, which displays a concentration of points denoting crystallographic orientation with respect to the {0001} plane in our study, is comparable to the display of sub-grain size calcite crystals in modern oyster shell samples (~27 microns for the our study and ~31 microns for Huerta et al., 2014). Together with this, we observed that the aspect ratios of calcite grains and local disorientation change within individual grains in the Cretaceous shell are similar to those in the modern shell studied by Huerta et al.(2014). Based on this evidence, we conclude that the Cretaceous shell carbonate crystals are well preserved and ideal for reconstruction of temperature using clumped isotopes.


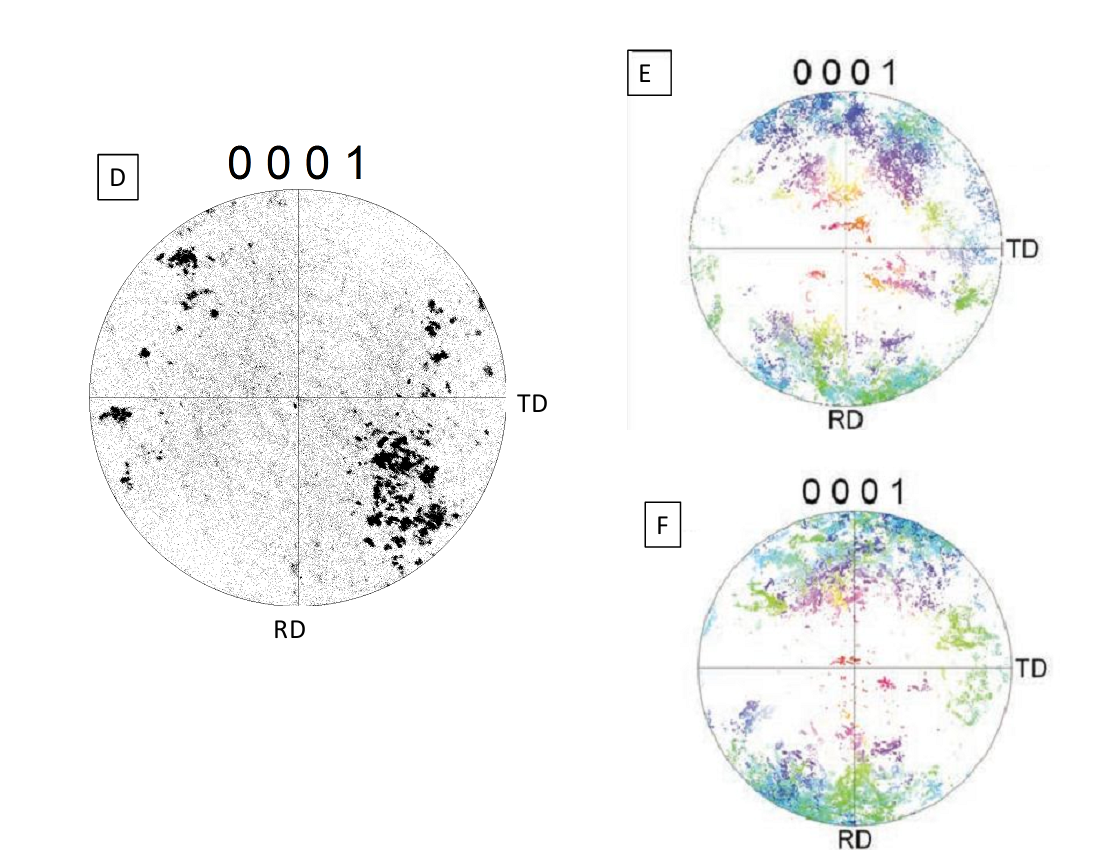

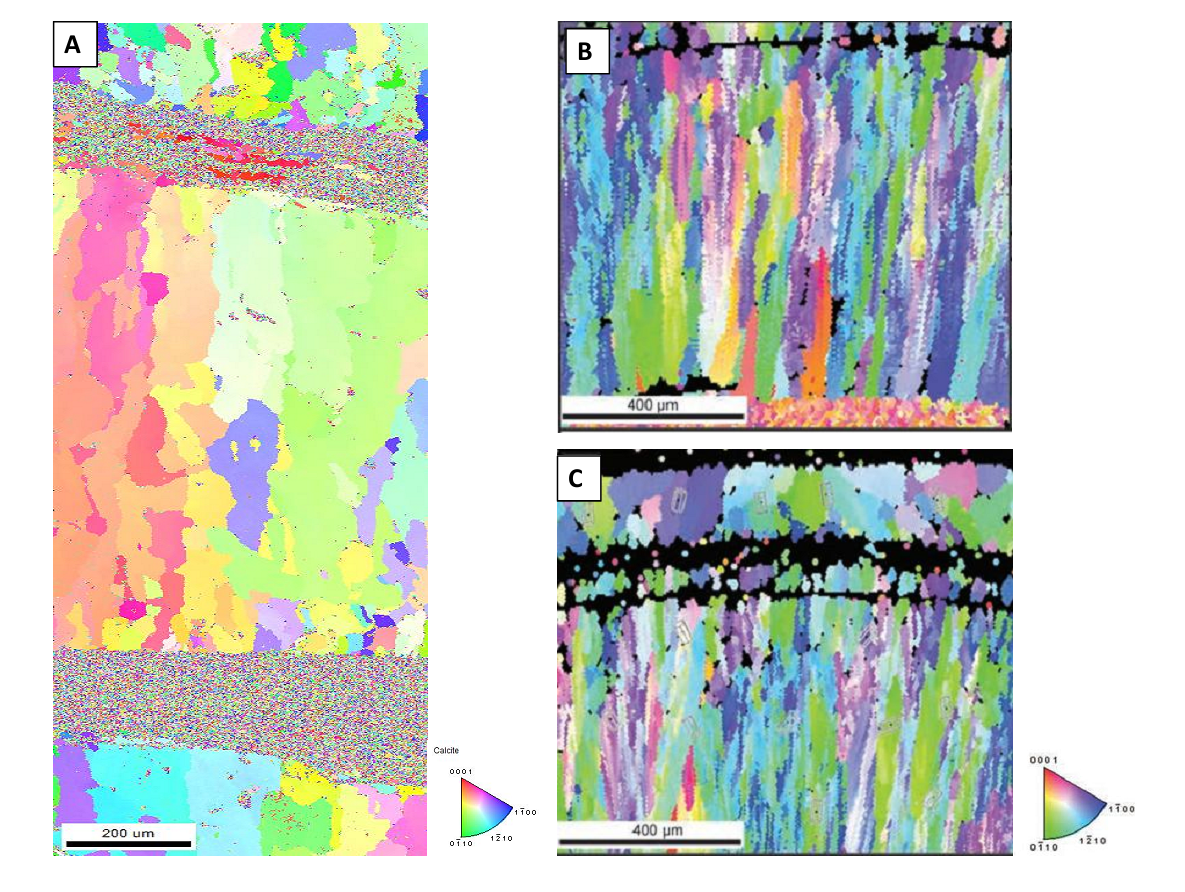


A1

A2

Figure S8: Crystallographic orientation map (A) of the Cretaceous *Phygraea* vesicularis shell investigated in our study, and the same shell investigated by Huerta et al. (2014). (B) Orientation at the interface between the nucleolus and the prism. (C) Orientiation at the transition of prisms towards the outer nacreous layer with colours representing different planes of calcite, as shown in the colour key. (D) Pole figure for the Cretaceous shell (this study) showing the orientation of crystals in reference to the {0001} plane, where A1 and A2 are equivalent to TD and RD of Huerta et al. (2014). (E) and (F) Pole figures of the shell investigated by Huerta et al. (2014) corresponding to panels (B) and(C)showing the orientation of crystals in reference to the {0001} plane.
